# Public Health Innovation through Cloud Adoption: A Comparative Analysis of Drivers and Barriers in Japan, South Korea, and Singapore

**DOI:** 10.3390/ijerph18010334

**Published:** 2021-01-05

**Authors:** Aarthi Raghavan, Mehmet Akif Demircioglu, Araz Taeihagh

**Affiliations:** Lee Kuan Yew School of Public Policy, National University of Singapore, Singapore 259772, Singapore; mehmet@nus.edu.sg (M.A.D.); spparaz@nus.edu.sg (A.T.)

**Keywords:** cloud adoption, public health, technology, healthcare, innovation, government, Asia

## Abstract

Governments are increasingly using cloud computing to reduce cost, increase access, improve quality, and create innovations in healthcare. Existing literature is primarily based on successful examples from developed western countries, and there is a lack of similar evidence from Asia. With a population close to 4.5 billion people, Asia faces healthcare challenges that pose an immense burden on economic growth and policymaking. Cloud computing in healthcare can potentially help increase the quality of healthcare delivery and reduce the economic burden, enabling governments to address healthcare challenges effectively and within a short timeframe. Advanced Asian countries such as Japan, South Korea, and Singapore provide successful examples of how cloud computing can be used to develop nationwide databases of electronic health records; real-time health monitoring for the elderly population; genetic database to support advanced research and cancer treatment; telemedicine; and health cities that drive the economy through medical industry, tourism, and research. This article examines these countries and identifies the drivers and barriers of cloud adoption in healthcare and makes policy recommendations to enable successful public health innovations through cloud adoption.

## 1. Introduction

Governments are increasingly adopting cloud computing around the world to deliver citizen-centric services [1,2]. It is an important information and communication technology (ICT) tool that enables the infrastructure upon which several e-governance initiatives can be based. Governments can host data and applications on the cloud (or the Internet) at a cheaper cost compared to spending for an extensive physical IT facility with recurring expenses. Cloud computing offers agility in terms of storage, management, processing, and sharing of data and/or applications, which can help governments to design innovative, cost-effective, and reliable solutions that are scalable and highly accessible [1,2,3].

Cloud adoption usually faces sector-specific drivers and barriers [4]. In terms of barriers, issues such as data protection and privacy, lack of interoperability, inadequate laws, cybersecurity concerns, maintenance requirements, national priorities and context, inadequate funding, and skills availability [5] can hinder efforts to adopt cloud computing on a large scale. However, governments are increasingly adopting cloud computing in key sectors such as healthcare, education, and financial services since it delivers in terms of cost savings, scalability, productivity [6], and open innovation [7,8,9,10]. While several studies discuss cloud adoption in public healthcare [11,12,13,14,15,16,17,18,19], there is lack of discussion on how cloud adoption leads to public health innovation, especially in Asia.

Motivated by this gap, this article aims to answer the following two research questions: *How can cloud adoption lead to public health innovation? What are the key drivers and barriers in the process?* To answer these research questions, the article maps the extent to which cloud computing has been adopted in the healthcare sector of Japan, South Korea, and Singapore, and how it has led to innovative healthcare practices.

The rest of this article is organized into five sections, starting with an introduction of cloud computing and why Asia offers a unique perspective. This is followed by a focus on cloud adoption trends in the healthcare sector and the reasons for choosing the specific country cases. The three country cases are discussed in detail with respect to their cloud adoption policies and how it has helped them develop innovative healthcare solutions. This is followed by a separate section that will discuss the key drivers and barriers in each case. Lastly, the article ends with an in-depth discussion consisting of policy recommendations, a conclusion, and potential future research directions.

## 2. Cloud Computing

According to the National Institute of Standards and Technology (NIST), cloud computing offers a “model for enabling ubiquitous, convenient, on-demand network access to a shared pool of configurable computing resources (e.g., networks, servers, storage, applications and services) that can be rapidly provisioned and released with minimal management effort or service provider interaction”. Some of the key characteristics of the technology are “on-demand self-service, broad network access, resource pooling, rapid elasticity or expansion, and measured service” [20] (p. 1).

Since 2006, when the term “cloud computing” was first introduced [21], it has grown into a USD 266.4 billion industry with an annual growth rate of 17 percent, with nearly 60 percent of all organizations expected to deploy the solution through an external cloud service provider [22]. Governments across the world are fast catching up [23] and allocating nearly 20.6 percent of IT budget at the local level and 22 percent at a national level for cloud adoption. This trend is expected to grow at nearly 17.1 percent through 2021 [24].

The role of cloud adoption in the public sector has been adequately explored in Europe and the United States, where it is a key underlying infrastructure that has enabled governments to store, share, and analyze data for bringing about improvements in existing services or innovate and create new services altogether [25,26]. In other words, cloud adoption leads to innovation in countries. However, governments adopting cloud are often faced with barriers. In Europe, for example, some of the key barriers have been “culture in countries, climate, legislation, economics and politics, IT staff shortage and feelings of uncertainty, fear and impatience” [27] (p. 1). It is the element of culture that drove this article to look at Asia for understanding this relationship better.

Research suggests that context plays an important role in cloud adoption, with countries in the Global North perhaps being at a different stage compared to those in the Global South [4]. While the USA and Europe have seen greater levels of government cloud adoption, Asian governments are just starting [28,29]. For instance, Japan, South Korea, and Singapore have been increasingly moving their public services onto cloud [30] and spending significantly towards building nationwide cloud infrastructure [3]. These countries also rank highly in terms of cloud adoption in the public and private sector combined. For example, Japan has a well-developed cloud infrastructure in place, South Korea has recognized cloud services as an integral part of the country’s Industry 4.0 strategy, and Singapore offers affordable cloud services [31,32].

Policymakers are increasingly finding it difficult to meet their citizens’ expectations in terms of the quality, speed, innovativeness, and ease of access to public services [33,34]. Cloud adoption addresses this challenge by decreasing the time, energy, and cost of design and deployment of public services [35]. It also makes public services accessible through mobile devices at any time and anywhere. The literature suggests that adoption of cloud computing can be an innovation in itself [36], although it can also be a tool to develop other innovations such as service innovation [37] and collaborative innovation [38]. Cloud service providers have studied the market extensively for commercial reasons and have identified areas where cloud adoption will enhance public sector capacities in specific sectors [39,40,41,42]. In Asia, governments have been proactively adopting cloud solutions [43], and many see this transition imperative, owing to existing and growing ICT infrastructure [44] and the increasing demand for citizen-centric services in the region [45].

## 3. Cloud Adoption in Healthcare

Healthcare expenditures are growing at a rapid rate in developing countries (6 percent annually) and developed countries (4 percent annually) [46,47,48]. In 2017, the World Health Organization (WHO) noted that healthcare expenditure in Asia will increase significantly over the next 10 years [49,50,51]. By 2050, Asia’s ageing population will further push demand [52,53], adding further stress on public healthcare facilities. Researchers observe that cloud computing saves cost [54], simplifies healthcare operations [55], and leads to innovations [56]. It is a technology that can offer considerable healthcare benefits [12,13,56] for both developed as well as developing economies [57]. It can save the cost of healthcare delivery through telemedicine [14,48], enhance access through cloud-based mobile applications [15,16], and improve efficiency and productivity through electronic health records [17,58,59].

To further explore this process, this study aimed to focus on healthcare systems in Japan, South Korea, and Singapore. These countries were chosen for the study owing to their advanced healthcare systems, initiatives for cloud adoption in the public sector, increased healthcare IT spending, and innovation capacity. Moreover, the three countries rank among the top five countries in Asia–Pacific on the basis of their Healthcare Access and Quality (HAQ) Index scores [60], with Australia and New Zealand being the other two nations [61]. In addition to healthcare advancements, a study conducted by Deloitte found that governments in these countries are increasingly considering cloud technology as a useful tool to bring down overall costs, enhance scale, and improve productivity and access of resources [62]. Furthermore, these countries have also emerged as the most innovative economies in Asia [63], and hence poise themselves as ideal candidates for this article’s research scope.

A report by the International Data Corporation (IDC) [64] suggests that healthcare IT spending, a key driver in overall healthcare expenditure in Asia, is expected to increase from USD 12.2 billion in 2019 to USD 14.9 billion by 2022, at a compounded annual growth rate (CAGR) of 7 percent. Interestingly, Singapore leads the region in terms of per capita healthcare IT spending, with the latest USD 37 million allocated to develop a dedicated healthcare cloud, named H-Cloud, aimed at moving patient health records to the cloud within 10 years [57]. Experts have observed that this expenditure is justified since it reduces the overall healthcare expenditure by nearly 12 to 17 percent and enhances the capacity of the sector to innovate [65].

Cloud computing is conducive to innovation in healthcare since it enables an organization to move from a capital-intensive model, with onsite server infrastructure, to an operation-based expense model using third-party service level agreements with cloud service providers (CSPs). This transition allows healthcare organizations to save cost since they need not build a dedicated and secure IT facility and instead spend only for essential applications, hosted on a remote server with industry-level security features [66]. Hospitals can use the cloud platform to offer innovative services, thus improving service outcomes in healthcare. Cloud computing can also help the sector to increase the scale of its services and provide last-mile access to healthcare through mobile applications [18,19].

## 4. Cases

### 4.1. Japan

Japan is one of the most advanced countries when it comes to healthcare access [67], quantity [68], quality [69], and efficiency [70]. As an aging society, Japanese healthcare faces a unique conundrum of increasing healthcare costs (per head and as a percentage of GDP) at a time when GDP growth has been decreasing consistently over many years [67]. Despite healthcare costs being lower in Japan compared to other developed countries, it has consistently added JPY 1 trillion to the government’s budget year after year (nearly 10.2 percent of GDP). This is because of the country’s aging population [70] and an early establishment of an extensive national health insurance system [71] that covers 70 percent of inpatient and outpatient costs [67]. While costs are controlled centrally [72], funding and service delivery have become fragmented at the prefectural levels due to demographic differences (with more elderly in rural areas requiring healthcare), lack of coordination between various institutions [73], and scarcity of doctors [74]. This has led to overlapping investments over the years and excessive deployment of resources [75,76]. Thus, integration of the various elements of healthcare service, optimization of resources, and a reduction in healthcare costs are some of the key challenges faced by the government [74,75].

Japan had achieved universal health coverage through social health insurance schemes since 1961. Since the 1970s, government policies in Japan consistently progressed from technology adoption in healthcare, which standardized and automated medical claims, to the automation of insurance claims and the development of electronic health records (EHRs) in the 1980s. This process led to increased information exchange between hospitals and the government. In the 1990s, information was standardized using a medical markup language (MML) and customized as per the preferences of each hospital. While this helped the government to collect data on insurance claims, it did not help in inter-organizational data exchange (i.e., exchange of patient information between hospitals) [77].

In 2001, the Government of Japan decided to adopt IT at scale across different sectors, including healthcare. To achieve this, they formulated national strategies (E-Japan Strategy I and II) that laid out the implementation guidelines in 2001 and 2003. Around this time, the Japan Association of Medical Informatics defined electronic medical record systems (EMR) for wider use. This new definition consisted of five levels of EMR adoption including (1) departmental, (2) inter-departmental, (3) hospital-wide, (4) inter-facility, and (5) inter-facility including care management information. Hospitals in urban as well as rural areas were expected to adopt at least level 3 for hospital-wide IT-based information exchange. This meant that hospitals should be able to collect and share information regarding patient’s complaint, symptoms, and treatment schedule electronically and across the hospital [76]. This threshold failed to encourage inter-facility information exchange, which is level 4. In addition, the government was especially careful to allow enough autonomy to healthcare organizations to adopt technology as per their available budget and expertise [77]. Over the next decade, policymakers in Japan pushed forward with policies that promoted the idea of information sharing across healthcare organizations to reduce costs and improve the quality of healthcare [77,78]. The Ministry of Health, Labor and Welfare (MHLW) published specific guidelines for the sector to increase overall technology adoption in 2001 and for utilization of digital information in medical care, healthcare, long-term care, and welfare sectors in 2007 [79]. A post-facto review of the guidelines suggests that while the Grand Design set the goal of the initial adoption of EMR across at least 60 percent of hospitals, only 10 percent of them adopted it by 2007 [80].

Moreover, many hospitals found that EMR adoption was expensive. While the government provided the initial funding for setting up IT systems, hospitals found that the cost of maintenance was not feasible for them in the long term [80]. Despite this, around 20.1 percent of hospitals had adopted EMR by 2011, which was a positive development [81]. The Japanese government continued to push for digitization of hospital systems with more specific national frameworks. For example, the Japanese cabinet’s “Declaration to be the World’s Most Advanced IT Nation” in 2013 stressed the importance of information sharing among medical and long-term care institutions for advancing the country’s healthcare sector [79]. Similarly, in the same year, consecutive versions of the Japan Revitalization Strategy emphasized on the need to integrate Industry 4.0 solutions in healthcare [82].

By 2015, the government had started to envision the development of public healthcare innovations through the adoption of emerging technologies [83]. In the same year, a “Working group on information and communication technology usage in the area of health care” was launched, which proposed the innovative concept of Person-centered Open Platform for Wellbeing (PeOPLe). According to this concept, health data generated throughout the life course of a person can be connected and integrated for use by both healthcare professionals and patients. The MHLW took up the recommendations of this working group to set up the “Administrative reform promotion office for health data” in 2017, which aimed to implement a model that would enhance seamless usage of data by both healthcare and long-term care professionals. Subsequently, steps were taken that enabled online claims systems, helped develop medical information databases, and encouraged hospitals to explore innovative ways of using health data for improved systems and better patient outcomes [79]. A major milestone was achieved in 2017, when the government legalized the use of big data (including cloud applications) in healthcare through the Medical Big Data Law, which detailed provisions for handling personal medical data and telemedicine, and covered online consultation within health insurance [84]. In the same year, the Personal Information Protection Act was passed, which established standards for careful handling of EHRs compared to other forms of personal information [85].

The PeOPLe platform is a key cloud-based innovation that is being built by that MHLW since 2016 as part of the Data Health Project (launched in 2015). The platform is aimed at providing “personalized medical care, improvement of medical services and the redistribution of resources” [86] (p. 276). It is expected to be operational by 2025 and will primarily collect personal medical information from hospitals, pharmacies, and the local government to build a database of the concerned person. These data can then be accessed and used by doctors to diagnose and prescribe treatments for the patient. While data collected through PeOPLe acts as the backbone, it will support the addition of other innovative solutions such as “self-recording lifelogs”, collected in real-time, that can further help in designing personalized healthcare for the aging population [86].

Another key innovation is the My ME-BYO Karte application software (ME-BYO is a Japanese word that means “pre-disease”) [87] (p. 1). It was developed as part of the Healthcare New Frontier (HCNF) policy package by the Kanagawa government in 2015, representing one of the fastest aging prefectures in the country [87]. ME-BYO is a concept that considers the “entire process of changes between mental and physical health and illness […] as continuous instead of dichotomous” [82] (p. 5). The project is built on data, in the form of personal health records (PHR), collected throughout the lifespan of an individual from birth to death, which will enable healthcare professionals to provide preventive care, thus increasing life expectancy of people. This can save the government nearly JPY 5 trillion a year, since preventive healthcare can decrease healthcare expenses and insurance payouts [75]. The platform requires data collected from different sources, about any given individual, to be in a continuous format to further add other solutions on top of it [82]. In February 2017, the concept was officially adopted in the Healthcare Policy document [87].

The ”My ME-BYO Medical Record Project” that evolved from My ME-BYO Karte application is a mobile application where people can record and monitor their health in real time, with the information always stored in the cloud for access, including in times of emergency [88]. It can help people to monitor their ME-BYO status on the app for dietary intake to prevent or delay the onset of illnesses [87]. The ME-BYO concept is an innovation that has provided a conceptual anchor for integrating advanced technologies (such as genomics, regenerative medicine, big data solutions, and robotics) in Japanese healthcare [88]. Taking it further, the Kanagawa prefecture has decided to model the “ME-BYO industry” to design advanced technological solutions that can contribute to economic growth while promoting the newly created products and services globally through international collaborations [74].

**Figure 1:**
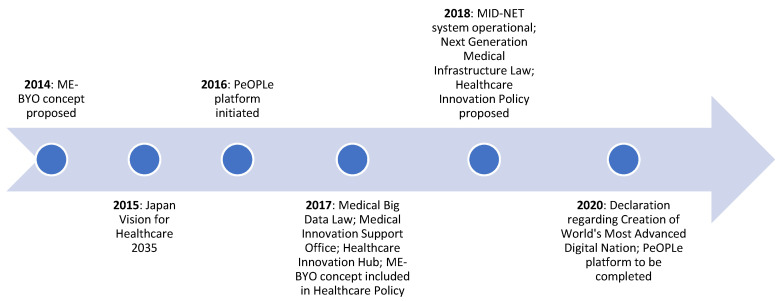
Policy trajectory from cloud adoption to public health innovation in Japan.

Figure 1 indicates the growing policy push towards technology adoption and tech-based public healthcare innovation in Japan. According to the 2035 Japan Vision for Healthcare announced in 2015, innovation is a key priority towards further developing the country’s healthcare infrastructure. The vision aims to make Japan an epicenter of health innovation. It also emphasizes the need to develop a national health database that can support telemedicine applications. By 2020, the government aims to build and utilize a healthcare network that links data using unique identifiers. Moreover, by 2035, it aims to use this data network for developing robust healthcare policies. The government also aims to establish a Bureau of Medical Innovation by 2020, which will drive efforts to evaluate health technologies and develop public healthcare innovations [83].

Some of the policy measures taken thus far include the setting up of the Medical Innovation Support Office in 2017, which supports research and development, regulatory approval, and expansion of innovations abroad. In July of that year, MHLW rolled out the Healthcare Innovation Hub, a networking facility for entrepreneurs. In order to have a legislative framework for public healthcare innovations, The Ministry of Economy, Trade and Industry (METI) (in 2018) proposed the Healthcare Innovation Policy, which aims to make use of cloud-based data and other emerging technologies to bring about new healthcare innovations in the country, with a focus on its ageing population [89]. Moreover, the government is also working on a sandbox framework to develop innovative digital healthcare solutions [90].

The government passed the Next Generation Medical Infrastructure Law in 2017, which came into force in 2018. It will allow hospitals to provide pooled anonymized patient data to companies to be accredited by the government [91]. Subsequently, the Government of Japan operationalized the Medical Information Database Network (MID-NET) in 2018, which opened patient data and medical and diagnostic information from 23 hospitals across 10 medical institutions to the pharmaceutical industry and academia. This is aimed at using anonymized data for medical research and development of new drugs. However, the system still fails to facilitate inter-organizational data sharing [92]. Going forward, the Japanese government aims to build on its rich national database to develop innovative public healthcare solutions using emerging technologies such as AI and the Internet of things (IoT) [84].

### 4.2. South Korea

South Korea is known for its rapidly aging population [93], technological advancement [94], and innovation [63]. Increasing healthcare expenditures, which currently account for 7.7 percent of GDP, is a significant challenge for the government, since this may lead to increased healthcare disparities in future as incomes stagnate [95]. While some observe that the impact of aging on healthcare expenditure has been relatively meager [96], it is probably because of a fast-growing GDP, which is expected to decline in the coming years [97], as the working class ages faster [93,98]. The healthcare system in the country is 90 percent privatized, with only 10 percent of hospitals being public [97]. As a result, out-of-pocket payment is high, sometimes reaching 50 percent for certain treatments [98]. Despite this, healthcare costs are covered mainly by the government through 100 percent payment through the Medical Aid Program and co-payments for the National Health Insurance Program (14 percent) and Long-term Care Insurance Program (20 percent). Although the country has a well-regulated and robust healthcare system, regional health disparities are observed, as 90 percent of doctors serve urban areas for 80 percent of the population who resides in urban areas [93]. The fragmented nature of the healthcare system and competition rather than coordination among healthcare institutions has led to inefficiencies in the system.

South Korea introduced its national health insurance system as early as 1977 and achieved universal healthcare in 1989 [95]. As a result, vast amounts of data have been generated, which have been regularly used to assess the efficiency of the healthcare system in the country [99]. In 1990, the Korean government announced its first 10-year Information Strategy Plan for National Health Insurance Service (NHIS), followed by a second version in 2001, covering all activities of the Ministry of Health and Welfare (MOHW), including hospital information systems and electronic data interchange [100]. While many hospitals were implementing electronic medical records (EMR), the system was legalized only in 2003, and by 2005, nearly 85 percent of healthcare clinics were implementing EMR [100]. In 2004, MOHW formulated a five-year plan to standardize the terminology and components used in hospital information systems and established the Center for Interoperable Electronic Health Records (CiEHR) aimed at developing a common information architecture. On the basis of this project, pilots were run in hospitals that saw a 5 to 12 percent reduction in annual healthcare costs [100].

In November 2007, the government funded a three-year Health Information Exchange (HIE) pilot program to understand the potential opportunities and challenges for the exchange of patient and clinical information between clinics and hospitals. It was implemented in 2009 after necessary updates to include the needs of private sector hospitals [100]. The information exchange was initially through paper, compact disk (CD), or memory stick, or online electronic exchanges [101]. Patient information was stored in the hospital’s own information management systems or on other domestic systems designed by private technology firms for healthcare purposes [102]. Although HIE was highly preferred by patients who perceived it to offer ease of access to quality healthcare services [101], it was expensive for smaller clinics since they had to maintain their own in-house IT infrastructure [103], especially in rural areas. As a result, there were disparities, with HIE rates being 58.1 percent between organizations [102]. However, NHIS strengthened the system by using a web-based model that helped outsource the procurement of required software applications, eventually reducing the cost incurred by clinics or community centers [100].

Despite the above changes, cloud adoption grew slowly, mainly because of regulatory barriers, data privacy concerns of citizens [100], and the concern of hospitals regarding potential market competition from large U.S. firms who could access patient data to target high-value customers [100]. However, in March 2015, the Government of South Korea passed the Cloud Computing Act, which laid the foundation of cloud adoption in the country [104]. Later that year, the Ministry of Science, Information and Communications Technology and Future Planning (MSIP) announced plans to move over 400 e-government services to the cloud by the end of 2015, and an additional 350 by the end of 2016, in order to achieve an improved public service and cost savings of approximately USD 32 million [105].

This encouraged Korean healthcare to undergo cloud adoption that was mostly domestic until then [102], and by 2017, EMR had been implemented in 93.6 percent of hospitals and 91.6 percent of clinics [102]. To achieve 100 percent adoption, Government of South Korea announced a three-year project called FEEDER-NET in 2018. As part of this project, the Ministry of Trade, Industry and Energy allocated USD 9.4 million to convert EMR data across hospitals into a common data module (CDM) and develop a cloud-based centralized platform that could work on the CDM network [106]. Forty-one hospitals have joined this initiative as of December 2018 [102]. In the same year, the government announced it plans to build a database consisting of genetic and biometric data of 10 million patients in collaboration with six hospitals. This was aimed at enabling the private sector use of this data for developing new solutions and products. A fund of USD 821 million was allocated for the development of “better data technology and revising data regulations to encourage further innovation and investment in big data solutions” [102] (p. 14).

U-health, also known as ubiquitous health, is a unique innovation of the Korean healthcare system. It includes the monitoring of patient health remotely through wearable devices [98]. The Korean market has seen massive use of wearable healthcare devices such as Apple Watch, Samsung Galaxy Gear, and Fitbit, which generate large amounts of personal healthcare data. This can allow healthcare professionals to monitor patient health in real-time [95]. The government too has been actively deploying a series of pilot projects under the umbrella of U-health. For example, the Institute of U-Healthcare, operated by the Seoul St. Mary’s Hospital at the Catholic University of Korea, is developing algorithms to filter data on blood glucose levels for patients. Research suggests that an innovation such as this can reduce physician time by 50 percent and reduce the cost incurred by the patient [98]. The fundamental idea behind U-health is to “maintain people’s health status” [98] (p. 12). The Korean government has been working on the concept since 2008 as part of the telemedicine service project and collaborating with local governments to deliver remote care. It aims to launch the technology further by converting it into a growth engine for the economy. For instance, the Ministry of Knowledge Economy (MoKE) in collaboration with the MOHW has been investing in “U-health city” in Wonju, which is expected to become a major hub for the medical technology sector. In addition, the Korean government also plans to develop and export its “IT-integrated hospital” model, which aims to bring together the country’s advanced clinical technology with IT solutions to promote medical tourism [98].

The second important innovation that has resulted from large scale data collection and the more recent cloud adoption is the Precision Medicine Hospital Information System (P-HIS). It was launched in 2017 by means of collaboration between the MSIP and MOHW. The project is aimed at developing a patient data platform for precision medicine. The platform will integrate a CDM for standardization of data that can then be used as part of P-HIS for implementing precision medicine in hospitals [107]. Since the platform is cloud-based and deals with such an extensive database of sensitive information, it was essential that the project strictly complied with patient privacy regulations [108]. As a result, the Korea University Medical Center (KUMC), which is leading the project, decided to collaborate with Switzerland-based Clinerion to use its Patient Network Explorer as an integral element of P-HIS. Clinerion’s “anonymized identification” technology enables the system to anonymize data at the source hospital and de-anonymize the same at the destination [109].

P-HIS is also part of a broader effort of the Government of South Korea to treat cancer, responsible for 30 percent of all deaths in the country. A fund of USD 83.34 million has been dedicated to promoting genomics analysis under the MOHW to address this challenge. In 2013, the Genome Technology to Business Translation Program was launched with an investment of USD 469 million over eight years to implement genomic medicine in hospitals. It works under the overall government mission named Genome Korea, which supports P-HIS by developing large biobanks at national and regional levels. The project will aim to extend precision medicine to complicated cancer treatments in the near future [102].

**Figure 2:**
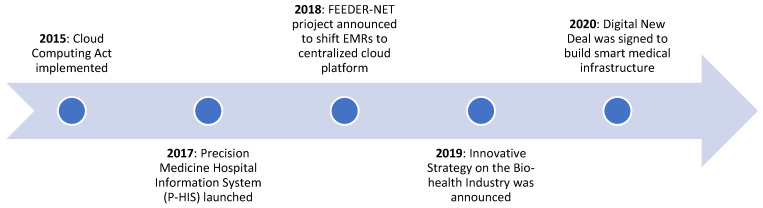
Policy trajectory from cloud adoption to public health innovation in South Korea.

As observed in Figure 2, the policy priority of the government is shifting from cloud adoption to healthcare innovation. Going forward, South Korea aims to invest heavily in healthcare innovation. This is apparent in the government’s “Innovative Strategy on the Bio-health Industry” announced in 2019. As per this policy, the government plans to establish five big data platforms, raise the government’s research and development investment to USD 3.6 billion, and improve regulations on review and approval of new healthcare innovations. As part of this initiative, the government plans to push towards the creation of an innovative ecosystem that will become an engine of growth for the economy [110]. To support this development, the government announced a budget allocation of USD 3.9 billion for emerging technologies such as artificial intelligence (AI) and digitization of healthcare infrastructure, among others [111].

### 4.3. Singapore

Singapore is a city-state with a rapidly aging population and is known for a robust healthcare system that is ranked highly in terms of efficiency and performance [112,113]. It is well known for achieving high health outcomes at a cost much lower than other developed countries (both as a percentage of GDP and per capita) [114]. However, healthcare expenditure has been increasing over the years, comprising nearly 4.4 percent of GDP in 2017 [115]. For example, between 2011-2013 government expenditure on healthcare increased by 46 percent from USD 4.1 billion to USD 6 billion [112]. The Government of Singapore ensures that citizens can pay for their own and their family’s healthcare expenditure through Medisave (the medical savings component of the Central Provident Fund (CPF)) [114], and that they are used only when essential [114,116]. The healthcare system is facing a shortage of skilled manpower and increasingly depends on foreign talent [116,117]. Hence, as the population ages, government insurance will likely be a key resource to fund healthcare [117] and will pose a significant financial burden for the country.

The Government of Singapore promoted a National Health Plan in 1983 with detailed plans for 20 years of implementation and the creation of Medisave. It is the savings-based national health insurance scheme. Over the years, the government also built other schemes such as Medishield and Medifund to cater to the growing healthcare needs of an ageing population [118]. In 1989, the Ministry of Health (MOH) contemplated a national medical information network, also known as MediNet. It was expected to increase connectivity in the healthcare sector through an integrated network that would link hospitals, clinics, medical practitioners, drug suppliers, and researchers with MOH and other government agencies [119].

Moreover, the network was expected to contribute to health surveillance, planning, and collection of comprehensive health data; lower costs; increase access; and help to improve the productivity of doctors who would be able to spend more time on patient care and research [119]. Several applications followed this initiative, including the National Patient Master Index (NPMI) (1994), the earliest version of the current National Electronic Health Record (NEHR). By 2006, NPMI was migrated to the Critical Medical Information Store (CMIS), which eventually led to the formation of EMRs [120].

Singapore has instituted three major healthcare clusters: SingHealth, National Health Group (NHG), and National University Healthcare System (NUHS) [113]. The first two initially developed their own EMRs, which were eventually integrated in 2004 into the Electronic Medical Record Exchange (EMRX), allowing them to view and share patient data online. Other information such as laboratory reports and medications were added digitally to EMRX in 2005. In the same year, a new EMRX was launched to extend its reach across more institutions, including immunization data, school health records (in 2006), and with increased access to community hospitals (in 2007). However, EMRX only allowed document exchange and lacked decision support capabilities [120].

NEHR was conceptualized in 2010 as part of the Intelligent Nation (iN2015) Masterplan—a 10-year plan to integrate the fragmented aspects of healthcare service delivery. In 2011, the Government of Singapore officially launched the NEHR system to create a central database of medical records accessible and shareable across different providers, as part of its “One Patient, One Health Record” strategic vision. It is owned by the MOH and managed by the Integrated Health Information Systems (IHIS) [120]. The IHIS launched the H-Cloud (Healthcare Cloud) in 2014 and migrated existing health data to it in 2015 [121]. In 2016, the IHIS standardized and consolidated over 500 applications used by the three layers of Singapore’s health systems, SingHealth, NHG and NUHS, on the H-Cloud [122]. In the first year, MOH allowed data contribution to be voluntary; however, contribution and access of data on NEHR was observed to be slow across public and private sector hospitals, with a very small percentage of people having accessed their medical data on the network [123]. It was expected that mandatory contribution to NEHR would accelerate cloud adoption and would be included in the Healthcare Services Bill to be passed in 2018 [124]. However, in July 2019, a cyber-attack led to nearly 1.5 million health records being compromised on SingHealth. As a result, the Healthcare Services Bill passed on 6 January 2020 excluded the provision for mandatory data contribution by public and private hospitals and instead put the system through a rigorous independent external review before being used as a national database [125].

Although adoption of NEHR was largely segregated across healthcare service providers in 2017 [123], by 2019, cloud adoption grew significantly, with nearly 89 percent of healthcare professionals in the country using NEHR. Being hosted on H-cloud, the platform currently supports over 50,000 healthcare providers across 9 public hospitals, 8 specialty centers, and 20 polyclinics and nursing homes to access patient data. It is expected to reduce costs by 55 percent and improve infrastructure availability to 99.95 percent by 2025 [122]. Since 2018, the Government of Singapore has been more focused on enabling conducive legislation to develop and deploy healthcare innovations in the country. As part of this effort, the MOH launched the Licensing Experimentation and Adaptation Programme, a regulatory sandbox, to “facilitate the development of innovative healthcare models in a controlled environment” [123] (p. 1).

HealthHub is an innovative online portal launched by the MOH in January 2016. The portal provided health data and information drawn from “multiple IT systems, including the NEHR System, National Immunization Registry, School Health System, and School Dental System” [120] (p. 150). HealthHub allowed access and sharing of personalized health records with family and healthcare providers. To attract people to use HealthHub, the MOH initiated a reward system that would give points to those who shared articles and events through the portal. Points accumulated over a certain period would be converted to food vouchers that could be availed at local grocery stores. As a result of this initiative, by 2017, HealthHub had 530,000 monthly page views, a total of 8.5 million page views, and nearly 84,000 unique downloads. Experts observed that the app helped increase health literacy, improved healthy habits, and nudged citizens to take a more proactive approach to maintaining a healthy lifestyle [120].

Smart Health Video Consultation (SHVC) is another innovation implemented across select public hospitals in 2017 to enable patients to consult their doctors post discharge from hospitals [113]. Examples of services include pharmaceutical consultation on select conditions, home care services, speech or hearing problems, post-stroke needs, communicable diseases, and cancer. The SHVC platform could be accessed using any computer or smartphone and includes two-factor authentication and end-to-end encryption to ensure the security of each video consultation session. The high-quality audio and video allow doctors to perceive patient conditions more accurately and help in cross-disciplinary consultations. The cloud-based solution allows file-sharing of documents and diagnostic images for reference during the consultation. The innovation allows doctors to save considerable time traveling to and from nursing homes, allows patients to get enough rest, helps caregivers to avoid absence from work, and reduces the overall exposure of patients to communicable diseases since they would have otherwise traveled to the hospital [120].

DigiMC is an innovation that was developed and launched by the Government Technology Agency of Singapore (GovTech) in 2019 to enable easier sharing of digital medical certificates between doctors, employees, and employers. The digital copy of the medical certificate is generated using existing systems and integrates through seamless backend integration. Digital copies can be generated online and be shared through SMS (short message service) messages (containing the link of the digital copy) to patients, who can then forward the same to their employers. Managed primarily by the IHIS, the SMS messages are backed up on commercial cloud services for later reference. Date of birth of the patient will be set for anyone to unlock the digital copy. In addition, a unique hash is generated for every digital MC, which can be used by employers to access the certificate on their system. GovTech is also considering integrating the system with HealthHub for one-stop access [126].

Conceptualized in 2010 and finalized in 2013, the Centre for Healthcare Innovation (CHI) was officially launched on 9 May 2019. It is one of the nine innovative projects to be launched by the MOH as part of the Health City Novena Master Plan 2030. The initiative is aimed at encouraging collaboration through a co-learning network that will involve all three public healthcare clusters (SingHealth, NHG, and NUHS) and other local and overseas partners. Currently, the center is building a national knowledge management system, with support from the IHIS, that will enable the healthcare clusters to interact and learn from each other. The online portal called CHI Learning and Development (CHILD) system is cloud-based and will facilitate access to information and best practices across healthcare institutions in the country [127,128,129].

**Figure 3:**
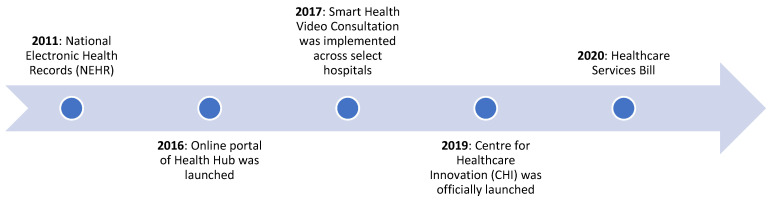
Policy trajectory from cloud adoption to public health innovation in Singapore.

As can be observed in Figure 3, the Singapore government aims to push for healthcare innovation while reconsidering its approach to healthcare management. Interestingly, the government has launched regulatory sandboxes for healthcare innovations since 2018 to support telemedicine and mobile medicine solutions [130]. Over the next five years, the government is expected to provide greater support for the adoption of emerging technologies in healthcare [131].

Table 1 provides a timeline of cloud adoption and healthcare innovations in Japan, South Korea, and Singapore. As seen from the table, all three countries demonstrate how sustained national policies have enabled cloud adoption and eventually public health innovations. However, the approach is different across countries. We observe that while Japan and South Korea have been more active in developing policy frameworks and institutional mechanisms for cloud adoption and public health innovation, Singapore has been active in terms of implementation. The latter was successful in systematically consolidating national health data, which eventually led to NEHR in 2011 and H-Cloud in 2014. The following section will explain the drivers and barriers to cloud adoption across the three countries.

## 5. Drivers of Cloud Adoption in Healthcare

### 5.1. Contextual Factors

#### 5.1.1. Demographic and Economic Factors

The three cases observed are all ageing populations that impose a significant economic burden on the healthcare system [67,70]. This is due to income stagnation over time as the working class ages faster [93,95,98]. This is a typical situation across the developed economies of Asia–Pacific that have attained advanced levels of development [52,53,132]. Adoption of cloud and other digital solutions aims to make healthcare data more accessible to a range of stakeholders, including healthcare workers, caregivers, insurers, and companies that develop new healthcare solutions for the ageing population. It also reduces the increasing burden on healthcare in terms of cost savings, in terms of per capita GDP, and resource optimization [14,17,48,54,58,133].

#### 5.1.2. Nature of the Healthcare System

The nature of the healthcare system is a contextual factor that can drive cloud adoption. Not all countries have a well-developed national health insurance program; however, those countries that have them can generate a large amount of health information, which aid the government in funding public healthcare [77,99,118]. The presence of national health databases makes cloud adoption more meaningful for governments since (1) it creates an infrastructure for data pooling and data exchange between hospitals and the government [91], and (2) it enables governments to evaluate their respective healthcare systems regularly and help them evolve, thus helping them appreciate the value of cloud adoption in healthcare early on [99,119].

#### 5.1.3. Technology Readiness and Innovation Mindset

Countries that want to adopt cloud need to have some level of technology readiness [134], including adequate funding [3,57], experience [30,31,32], and institutional mechanisms [76,100,120]. These elements drive cloud adoption from planning to implementation. A few other factors that contribute to technology readiness are innovativeness and optimism [135]. At a policy level, the countries studied are highly innovative and have high aspirations to grow into digitized societies [136].

### 5.2. Policy Factors

#### 5.2.1. National Policy Frameworks that Promote Digitization of Healthcare

National IT policies are key drivers of cloud adoption in all three countries. They set the vision and the path along which a government will eventually progress to digitized healthcare, among other sectors. Beginning with broader technology frameworks [76,79,82,100,120,131], countries may eventually progress to develop frameworks for digitization of healthcare [98,110,119,127,128,129].

However, policy frameworks also need to be realistic. In the case of Singapore, the Ministry of Health (MOH) first undertook a feasibility study with the National Computer Board (NCB) to attack implementation bottlenecks beforehand. With a clear vision to integrate healthcare information across the island nation, the government was able to drive cloud applications using the NEHR backbone [120].

Policy frameworks set the vision for the government to implement policies that favor technology adoption, reduce barriers in the process, and support the transformation financially and technically to evolve over a long period [118]. Each of the countries revisited these frameworks from time to time and revised them to meet their evolving needs [76,100,131]. They sustained in their efforts to digitize healthcare [77,78,79], allocate enough resources [57,102], and engage stakeholders to scale initiatives across public and private healthcare [100].

#### 5.2.2. Cloud-Specific Policies

Governments have acknowledged that adoption of cloud will be inevitable going forward [43,44]. Hence, as seen in the three cases, policymakers have implemented cloud-specific policies to better regulate this rapidly evolving technology [84,91,104,120]. Cloud-specific policies helped legalize cloud adoption and helped the government guide its adoption across sectors, particularly in healthcare [84,104,121]. It also helps build trust across public and private healthcare sectors and generates opportunities for greater collaboration [91,92,100]. Other types of cloud policies include those related to storage, standardization, sharing, privacy, and security of data on the cloud.

##### Data Standardization

Health data are different from other forms of data. They have a specific purpose in the healthcare sector and are meant to be accessible and readable for different kinds of healthcare service providers [108]. Data standardization can be challenging because it may not necessarily allow for inter-organizational data exchange [77]. However, if standardization is planned well, with enough time for its adoption, then it can be an important driver for cloud adoption [100,120].

##### Data Storage

Data storage policies are aimed at guiding hospitals on who can store health data and what precautions need to be taken with respect to such data. A policy such as this can direct healthcare organizations to select a suitable cloud service provider (CSP) who offers reliable security features, executes an agreement that ensures the CSP’s proper handling of confidential health information, and obliges the hospital to regularly supervise the CSP [137]. It can also guide public and private hospitals and CSPs on how such data should be handled and where it can be stored [107,138]. Once there is enough clarity on this issue, healthcare organizations usually find it more comfortable to adopt and operate in the cloud [108].

##### Data Privacy

Strong data privacy regulations can drive cloud adoption healthcare [134]. While it is challenging to design such policies, governments usually revise existing policies to tailor them to the privacy needs of the healthcare sector [85]. Since identifiability of health data is a key reason for data privacy concerns, governments have taken the approach of anonymizing health data to make it safer for sharing across the healthcare ecosystem [91,92]. In addition, clear guidelines for data handling, and an independent authority overseeing enforcement of privacy regulations can be crucial in securing health data [85]. For example, in Singapore, the Personal Data Protection Act (PDPA) (2012) governs the access to and collection, use, disclosure, and care of personal data stored in electronic and non-electronic forms. The law requires healthcare institutions to ensure adequate safeguards for data, especially when patient records are shared from one provider to another, within the country or overseas [120,139].

##### Cybersecurity

While cybersecurity law can be a strong measure, governments can deploy industry best practices to enhance the security architecture around health data [18,140]. A strong cloud security architecture is essential to ensure that healthcare operations are conducted smoothly while ensuring that sensitive health data are protected [18]. Other measures that can be taken include setting up of dedicated cybersecurity institutions that can monitor threats in real time, a framework for sharing cybersecurity information, and licensing of cybersecurity service providers [141]. Building adequate safeguards to protect national healthcare database from cyberattacks is a crucial step to build the necessary trust for greater cloud adoption in the sector [142].

For example, in Singapore, the National Registration Identity Card (NRIC) has been added as an additional layer of safeguard for health records. Singapore Personal Access (SingPass), launched in 2003, acts as an online identity card that allows access to digital services offered by more than 60 government agencies. The same has been linked to HealthHub for allowing access to medical records online and through mobile applications [143]. Such measures significantly reduce cyberthreats in the healthcare sector.

#### 5.2.3. Incentives for Cloud Adoption

Incentives can be effective drivers in increasing cloud adoption in healthcare [144]. As observed in the cases, most hospitals and clinics found the initial capital expenditure and the long-term maintenance of such systems to be expensive [80]. However, all three countries offered direct or indirect incentives to hospitals to encourage them for cloud adoption. Japan offered financial support for establishing IT systems [80], whereas South Korea offered small incentives over a longer duration of time [103]. Singapore, on the other hand, incentivized CSPs to register with the Multi-Tier Cloud Security Standard for Singapore (MTCS SS), established in 2013. MTCS then enabled cloud users to access the self-declaration submitted by CSPs and offered a clear comparison of their capabilities to enable cloud users to choose the best service provider. While this was not specific to the healthcare sector, this approach enabled cloud adoption since it built public trust on MTCS certification of CSPs [145].

While direct incentives such as autonomy and incentives for healthcare providers and CSPs played an important role in cloud adoption [80,103,123], there were indirect incentives that contributed to the same. For instance, in South Korea, private hospitals feared competition for health data from rivals as a barrier for cloud adoption [100]. However, the South Korean government’s overall push for increased cloud adoption in public services created a positive environment and encouraged private healthcare institutions to do the same [102].

The broader incentive for cloud adoption is that, in addition to saving costs and increasing efficiency in healthcare, it can aid in long-term economic growth of countries [146]. Policymakers are increasingly realizing this and hence continue to embrace the idea of adopting emerging technologies such as cloud computing in healthcare. This strategy is expected to not just help them to solve their current problems but will also enable their societies to be future-ready [110,127,128,129,136,147].

### 5.3. Human Factors

#### Public Trust and Acceptance

Public trust in government initiatives is a crucial factor when it comes to the success of cloud adoption and innovative digital solutions in healthcare [148]. Public acceptance of cloud-based solutions such as NEHR in Singapore [123] and HIE in South Korea [101] can be a key driver for the government and the healthcare sector to actively drive cloud adoption. Interestingly, however, policies can drive public acceptance as well. An example is how Japan has envisioned “social acceptance” as a key idea in its Society 5.0 policy document. This approach is expected to better integrate advanced technologies with society in general [149].

Table 2 provides a summary of the drivers for cloud adoption in healthcare.

## 6. Barriers to Cloud Adoption in Healthcare

### 6.1. Contextual Factors

#### 6.1.1. Healthcare Status

Lack of basic healthcare infrastructure, including universal health coverage and national health insurance schemes, can be challenging for cloud adoption [6]. These are systems that help centralize data in public healthcare systems and thus make cloud adoption more practical. It is also important to consider the distribution of large, medium, and small hospitals (or clinics) in each country context. Research suggests that small and medium hospitals adopt cloud more slowly and over a longer period compared to large hospitals. Small health clinics find the transition most difficult [81].

#### 6.1.2. Technology Readiness

Discomfort and insecurity with technology are aspects of technology readiness that can act as barriers for cloud adoption. Some aspects of negative technology readiness can include safety concerns, concerns about the negative consequences of technology, and a need for assurance [135]. These issues can be present at the policy level; organizational level [150]; or at the level of end-users [151], i.e., patients in this case, and can be significant barriers for cloud adoption. For instance, after the 2018 cyberattack in Singapore, there were increasing concerns regarding the safety of cloud-based solutions among policymakers. As a result, they decided to hold an independent review of the NEHR before deploying it at a large scale [125].

### 6.2. Organizational Factors

#### 6.2.1. Cost of Cloud Adoption

It has been observed that the high cost of digitization is a key barrier to cloud adoption in healthcare in general, especially for small healthcare clinics or hospitals with a limited budget [79]. Although research suggests that the initial high cost of cloud adoption can be recovered through increases in the overall productivity of the system and optimization of resources [83], an alternative view suggests that, in certain cases, gains from cloud adoption might not be enough to move from existing systems to the cloud in healthcare [152]. This is explained by the fact that some parts of the in-house IT operating costs will continue, even after cloud adoption, including costs associated with maintenance, training, IT staff salary, utilities, supervisory staff salary, hiring, bandwidth, and other associated tasks. As a result, “a systematic ‘people, process, and policy’ transformation is required after cloud adoption to control these cost elements and thereby gain the full benefits of cloud computing” [152] (p. 215).

#### 6.2.2. Technical Expertise

Lack of technical expertise in organizations is, in general, a barrier to cloud adoption in the public sector [153]. However, this can also be the case in the healthcare sector [154]. The health sector needs to overcome this in order to effectively deploy and operate cloud solutions.

### 6.3. Policy Factors

#### 6.3.1. Lack of Data Standards

While South Korea and Singapore have been relatively successful in standardizing healthcare data for uniform cloud adoption, Japan has been lagging. As of 2017, EMR adoption rate in the country has been a mere 34.4 percent [6], which is quite low despite several initiatives to promote cloud. In 2013, the Japanese government established a Social Society and Tax Number System (SSTN) and set up a committee that considered a variety of scenarios whereby institutions and activities related to healthcare data could be linked [74]. It was, however, not widely successful, owing to data privacy and security concerns. Thus, in 2015, the Japanese government introduced the My Number system, which has recently been linked to the national health insurance system [155]. Despite all these efforts, data standardization is still a work in progress in Japan.

Existing literature suggest that standardization of data allows for greater interoperability, which in turn increases cloud adoption [156]. Some of the factors that affect interoperability include language, which can at times lead to terminology- and translation-related issues [156], and the design of the EHR system itself, which can make documentation of complex medical cases more difficult for healthcare professionals [157].

#### 6.3.2. Strict Data Storage Requirements

Lack of clear and flexible legislations regarding cloud data storage can hinder cloud adoption. Governments are increasingly realizing the importance of data, especially health data, which consists of highly sensitive information. While productive use of this data can aid in healthcare innovation, it can be misused if it falls into the wrong hands [158]. In South Korea, strict data storage regulations called for health data to be stored within the hospital premises. However, this hindered largescale cloud adoption owing to several hospitals and clinics lacking data storage resources. However, recently, the Government of South Korea changed regulations to allow EMRs to be stored off-premise and onto the cloud. This has now allowed healthcare institutions (nearly 100 hospitals, medical centers, and other facilities) to store patient data on the cloud [108], although it is still mandatory for health data to be stored within South Korea [107].

#### 6.3.3. Data Privacy Concerns

Lack of strong legislations to ensure data privacy can be a significant barrier to cloud adoption [135]. It hinders public trust of the system and nation-wide adoption. For instance, the Personal Information Protection Act (PIPA), enacted in South Korea, fails to distinguish explicitly between personal information and health information. Moreover, there is an overlap between the Medical Services Act (2001) and PIPA, which has created ambiguity in terms of providing the necessary protections to sharing of EMRs between organizations [159]. Overcoming such gaps can be crucial in achieving cloud adoption in healthcare since it helps build the trust of citizens and healthcare organizations in the technology.

#### 6.3.4. Weak Cloud Security Infrastructure

Public healthcare institutions are not actively considering cloud security models, and as a result they are increasingly vulnerable to cyberattacks [160]. A key reason for this is that enhanced cybersecurity entails financial and opportunity costs. Institutions find it difficult to allocate adequate resources for the purpose within their limited resources [161]. This is a significant barrier for cloud adoption as any potential threat may deter ongoing policy efforts if not handled well [125].

Table 3 provides a summary of the barriers to cloud adoption in healthcare.

## 7. Discussion

**Figure 4:**
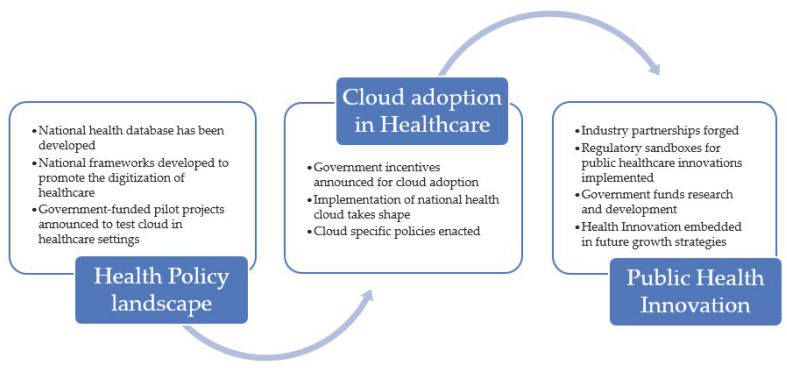
Processes that drive cloud adoption in healthcare to public health innovation across the three cases.

Cloud adoption in healthcare is strongly linked with public healthcare innovations. Figure 4 presents an overview of this process.

However, in addition to policy processes, there are hospital-level factors that play a critical role in this transition [161]. While hospitals that receive adequate funding and technical support are more adept at cloud adoption, it has generally been observed that inter-hospital and intra-hospital factors are more complex [77] and can vary from one country to the other. Existing research also suggests that while cost-effectiveness is a critical factor for cloud adoption at a policy level, data security and privacy are key determinants at the hospital level [162]. Similarly, cost can be a larger determinant when comparing large and small hospitals and/or clinics, as observed in the cases of Japan [80] and South Korea [102].

Another key observation is that the drivers and barriers for each of these innovations are different. This finding has important implications for studies on innovation because there are many sources of innovation. For example, in the case of Japan, Kanagawa prefecture’s governor, Yuji Kuroiwa, was a key proponent of the ME-BYO concept of healthcare innovation [87]. On the other hand, in the case of Singapore, several public health innovations are institutionally driven [120,126]. Therefore, the findings of this study provide evidence that sources of innovation can vary from one context to the other [163,164,165,166,167].

Meanwhile, barriers to cloud adoption can be drivers for innovations. For example, contextual barriers such as ageing population, increasing cost of healthcare, and resource fragmentation have been effectively used as drivers for cloud adoption in the three cases. Similarly, governments can transform slow cloud adoption rates by converting client server-based model of health data to a web-based model, thereby reducing IT costs for healthcare institutions [100]. These findings are consistent with early studies finding that, in fact, barriers can be positively associated with the implementation of innovation [168,169,170,171]. For example, barriers can increase managers’ awareness of obstacles, and enable them to develop strategies to overcome those barriers [169,171]. More specifically, Torugsa and Arundel (2016, 410) argue that “Instead of measuring impediments that entirely prevent or deter innovation, … barriers measure the awareness of public employees of problems that must be solved in order to innovate, or what D’Este et al. (2012) describe as the ‘revealed’ effect of barriers.” Thus, policymakers should look at barriers as unique opportunities for innovation [166].

These observations are particularly relevant to developing countries such as China, India, Malaysia, and Thailand, which are witnessing cloud adoption in the private healthcare sector [172]. Governments in these countries are also considering ways to translate the benefits of cloud to public healthcare at scale [173,174,175,176], which may potentially lead to innovative healthcare solutions in future. Governments across the world can use the findings to develop their own policy approaches to adopt in healthcare, navigate through the contextual drivers and barriers, and lay the groundwork for successful implementation eventually. On a broader scale, this article aims to inspire developing countries to consider the benefits of cloud adoption in the long run to solve some of the pertinent and increasingly complex challenges that are evolving in healthcare.

Finally, cloud adoption has come as a significant boon for managing pandemics such as COVID-19 (CoronaVirus Disease 2019) on a global scale. COVID-19, or coronavirus, was first reported in the city of Wuhan, China [177,178,179], eventually spreading across the globe, killing thousands of people, distressing economies, and negatively affecting healthcare capacity and systems [180,181]. As the pandemic spread rapidly in 2020, most governments were faced with little time to respond [182]. This led to a series of procurement, mass testing, policing, and digital initiatives at a scale never seen before. Many governments turned to using healthcare data to effectively curb the spread of the disease [182]. For example, South Korea and Singapore have developed and deployed contact tracing mobile apps to implement social distancing and track the movement of quarantined people in the country [177,183,184]. Experts observe that existing healthcare infrastructure, IT expertise, and the level of trust between citizens and government are vital factors for the success of these solutions, as observed in South Korea [185]. More importantly, the value of cloud computing is best reflected through the pace at which it has abetted a global healthcare response to the pandemic from multilateral institutions, private technology firms [186], and academic and research institutions [187], which in turn guided governments (central and local) in terms of best practices that can be used to respond to the pandemic in a timely manner.

Interestingly, the COVID-19 pandemic catalyzed a renewed interest among patients and healthcare providers for telemedicine applications in the concerned countries [136,184], including the use of AI in remote health diagnosis [184]. The private sector too saw a wave of health innovations that offered cloud-based telemedicine services [188]. As a result, the governments not only eased existing regulations on remote medical treatment but also passed new regulations to promote digital-based smart medical infrastructure that would protect medical staff and provide more convenience to patients [147]. More importantly, the pandemic came as a wake-up call for government agencies that had failed to digitize fast enough [136]. The situation also increased the political will for cloud adoption and public healthcare innovation [147]. For instance, in July 2020, the Japanese government signed the declaration regarding the Creation of World’s Most Advanced Digital Nation to digitize the entire Japanese society in the coming years [136]. Going forward, investments in AI and IoT are expected to enable new healthcare innovations that will improve the resilience of healthcare systems to deal with global pandemics [147].

### 7.1. Policy Recommendations

#### 7.1.1. Undertaking Sustained Policy Approach for Cloud Adoption

Far-sighted national information technology strategy and consistent policy approach thereafter can help governments achieve cloud adoption in healthcare. Early adoption is also useful since it can aid in developing nationwide healthcare records and design healthcare innovations on the basis of such information backbone [121,189]. Governments have the capacity and play an important role in adopting and promoting cloud adoption. In fact, “governments play a crucial role in promoting CC [cloud computing] adoption. Without timely and effective promotion strategies, a country and its business sectors may become trapped at a level of CC adoption that is low” [3] (p. 32). In addition, design and implementation of cloud adoption in healthcare should address the specific requirements in the sector, including policies that address needs for standardization, storage, privacy, and security of sensitive healthcare data.

#### 7.1.2. Incentivizing Cloud Adoption for Healthcare Institutions

Given the cloud adoption offers significant benefits to healthcare, governments should provide incentives to healthcare organizations to actively adopt cloud. Many studies including both the government and private sector consistently demonstrate that providing incentives increase innovations in any government and private organizations [190,191,192]. However, policymakers should specifically focus on medium and small hospitals, including clinics, which experience the slowest pace of cloud adoption. Incentivizing them can fasten the pace of adoption and make health data richer and more accessible at a national level.

#### 7.1.3. Standardizing Health Data for Largescale Adoption

Health data should be standardized on the basis of international best practices. This is important since standardized data enables healthcare providers to access it on a timely fashion, thus improving treatment and eventually health outcomes for patients. Governments should allocate enough resources, in terms of experts, institutions, and funding, to this process. This will allow health data standard to be developed with relevance to the country context while addressing the inherent complexities of the medical field.

#### 7.1.4. Enabling Flexible Data Storage Policies

The government should adopt data storage policies by keeping in mind the requirements of the healthcare sector. Many countries in Asia, including the focus countries, have taken a strict approach and enforced strong on-premises or in-country data storage norms. However, governments should consider the impact of such policies on the growth of cloud adoption, particularly from the perspective of healthcare organizations and cloud service providers. CSPs should be allowed enough flexibility to offer affordable and accessible cloud services to clients in the public healthcare sector. Restrictive data storage policies can affect their cost structure and hence limit the range of services that they can otherwise offer.

#### 7.1.5. Strengthening Data Protection

Governments need to enact strong data protection policies that specifically identify health data and address the concerns that may arise from its sharing. Protection of health data is an important issue for healthcare institutions from an ethical perspective. It is also a core concern as any violation of patient privacy can leave a deeply negative impact on the reputation of the concerned healthcare organization. Data anonymization is a good measure; however, governments must also explore the use of emerging technologies such as blockchain to ensure that data are only accessible to those intended.

In cases where there are gaps in data protection laws, governments should make an effort to amend laws on the basis of expert opinion. A good example is how South Korea updated its data privacy legislation by announcing the Guidelines for the De-Identification of Personal Information (GDPI) (2016), which essentially allows sharing of data in its anonymous form for big data applications [159].

#### 7.1.6. Improving Cybersecurity Architecture of Health Cloud

Cybersecurity threats are increasing in the healthcare sector and can pose significant challenges to cloud adoption going forward. Policymakers must take cybersecurity of cloud-based healthcare records very seriously. Singapore’s experience with a national data breach in 2018 is an example of the extent of damage cyberattacks can cause to digitization efforts. Existing literature points towards an adaptation-oriented approach that can help policymakers address technological risks associated with emerging technologies such as the cloud [193]. Governments should proactively develop robust policies that ensure data privacy and security in the healthcare sector. This can be especially beneficial in building public trust in the system and enable greater engagement with the private sector, which in turn can guide innovative healthcare solutions.

#### 7.1.7. Encouraging and Supporting Public Healthcare Innovations

Governments should adopt future-centric policies while focusing on emerging healthcare challenges in their respective countries (including ageing populations and rising costs) and regularly monitor new developments and risks with respect to cloud computing and other emerging technologies. With the right kind of legal framework, encouragement, and support in place, the government can play a huge role in the promotion of unique healthcare innovations, which is consistent with other studies on innovation [194,195]. As observed in the cases, healthcare innovations grant the country-of-origin strategic benefits with respect to economic growth. Healthcare innovations can help countries build new brands (such as ME-BYO), new healthcare areas (such as U-Health), or even new opportunities for international collaboration for research in healthcare solutions (such as Health City Novena in Singapore).

### 7.2. Study Limitations and Future Research Directions

This study is primarily limited by the range of information available through secondary sources. However, future research can explore this topic in greater depth, potentially at an institutional level, to extract rich contextual information on the factors that contribute to healthcare innovations as a result of increased cloud adoption. While doing so, researchers may choose to conduct interviews with policymakers to understand their approach to public healthcare innovation, including the main sources/actors for innovation, how innovations are developed, barriers to innovation, and how these barriers are overcome. This topic can also be expanded further by studying countries such as China, India, Malaysia, and Thailand, which are rapidly adopting cloud in public and private healthcare. Future studies can also consider analyzing cloud adoption in other sectors such as education and financial services. Lastly, it would be interesting to explore what healthcare institutions and governments are doing to overcome cybersecurity threats going forward.

## 8. Conclusions

Existing literature covers cloud adoption in healthcare from a multitude of perspectives and from various country contexts. There is broad consensus that technology adoption is linked to innovation in the healthcare sector. However, there are very few studies that explore this connection in the public sector, especially in the Asian context. The article’s unique contribution lies in its exploration of the policy processes behind cloud adoption and healthcare innovation in three Asian countries. This article makes contributions that could benefit academics and practitioners. First, it identifies the drivers and barriers to cloud adoption across Japan, South Korea, and Singapore. Second, it identifies the process by which cloud adoption led to public health innovation in the concerned countries. Third, it makes important policy recommendations that can enable cloud adoption and public health innovation in other countries. Overall, this study aims to inform policymakers, researchers, industrial experts, and students on how governments in the region approach cloud adoption in healthcare and the process by which it may translate to public health innovations.

More specifically, this article identifies the drivers and barriers of cloud adoption by categorizing them into contextual, policy, organizational, and human factors. On the basis of these factors, the article outlines seven recommendations for policymakers who are willing to adopt cloud in the healthcare sector. These recommendations sustained policy approach, incentives for healthcare organizations, standardization of health data, implementation of flexible data storage policies, strong data protection legislation, improved cybersecurity architecture, and support for public healthcare innovations. Future research can explore the process of cloud adoption and healthcare innovations in developing country contexts in Asia and beyond. It will potentially enhance our understanding of the role of context in technology adoption and public sector innovation. At a time when the COVID-19 pandemic has greatly increased policy interest in digital healthcare, this article presents timely findings for policymakers to build innovative digital healthcare systems.

## Figures and Tables

**Table 1:** Timeline of cloud adoption and healthcare innovations across the three countries.

Year	Japan	South Korea	Singapore
1961	National Health Insurance system completed		
1977	Standardization and automation of medical claims processing in 1970s	National Health Insurance Act	
1983			National Health Plan
1989	Development of electronic health records (EHRs) in 1980s	National Health Insurance Service	MediNet
1990	Development of medical markup language (MML) in 1990s	10-year Information Strategy Plan for NHIS	Central Claims Processing System Physician Data Query System for Cancer
1994			National Patient Master Index (NPMI)
2001	E-Japan Strategy Grand Design for Informatization of Healthcare Field	Second 10-year Information Strategy Plan Medical Services Act	
2003	E-Japan Strategy 2 Electronic medical records (EMR) systems defined	Electronic medical records legalized Centre for Interoperable Electronic Health Records	Singapore Personal Access (SingPass) Electronic Medical Record Exchange (EMRX)
2005			EMRX data expanded
2006	New IT reform strategy		NPMI migrated to Critical Medical Information Store EMRX data expanded
2007	Grand design for information utilization in medical care, healthcare, long-term care, and welfare sectors	3-year Health Information Exchange (HIE) Program pilot	EMRX expanded to community hospitals
2008		Concept of U-Health emerged	
2009	i-Japan Strategy 2015 launched National Database of Health Insurance Claims and Specific Health Checkups of Japan	HIE updated with needs of private sector addressed	National Electronic Health Record (NEHR) initiated
2010	A New Strategy in Information and Communications Technology		NEHR conceptualized Centre for Healthcare Innovation (CHI) conceptualized
2011		Personal Information Protection Act (PIPA) enacted	NEHR officially launched
2012			Personal Data Protection Act (PDPA) enacted
2013	Declaration to be the World’s Most Advanced IT Nation Japan Revitalization Strategy Social Society and Tax Number (SSTN) System was established	Genome Technology to Business Translation Program	CHI finalized
2014	Healthcare New Frontier (HCNF) policy package Revision of Personal Data Protection Law Committee to decide on the use of SSTN System in healthcare		Application of the PDPA for the healthcare sector H-Cloud launched
2015	Japan Revitalization Strategy Updated Working group on information and technology usage in the area of healthcare The Japan Vision: Health Care 2035 Data Health Project launched	ME-BYO registered as a trademark Cloud Computing Act passed Ministry of Science, Information and Communications Technology and Future Planning (MSIP) announced plans to move over 400 e-government services to the cloud	Information Protection Measures for Vitalization of Cloud Services announced Health data from across hospitals migrated to the H-Cloud
2016	Japan Revitalization Strategy Updated Person-centered Open Platform for Wellbeing (PeOPLe) started Healthcare Business Contest by METI	Guidelines for the De-Identification of Personal Information (GDPI) MSIP to move 350 more e-government services to the cloud	IHIS standardized applications across health clusters HealthHub launched
2017	Medical Big Data Law Next Generation Medical Infrastructure Law passed Administrative reform promotion office for health data Medical Innovation Support Office Healthcare Innovation Hub ME-BYO included in Healthcare Policy Personal Information Protection Act	Precision Medicine Hospital Information System (P-HIS) launched	Smart Health Video Consultation (SHVC) implemented
2018	Next Generation Medical Infrastructure Law comes into force Medical Information Database Network (MID-NET) Japan Healthcare Innovation Policy (draft) Sandbox framework to promote health innovations	FEEDER-NET announced Common Data Module (CDM) implemented Government announced a plan to build a national database of genetic and biometric data	Cyberattack on SingHealth Licensing Experimentation and Adaptation Programme Personal Data Protection Commission (PDPC) established
2019	2019 Growth Strategy	Innovative Strategy on the Bio-health Industry	DigiMC launched CHI launched CHI Learning and Development (CHILD) system launched
2020	PeOPLe expected to be completed Declaration regarding Creation of World’s Most Advanced Digital Nation	Digital New Deal policy	Healthcare Services Bill passed

**Table 2:** Drivers.

Drivers for Cloud Adoption in Healthcare
Contextual factors ○ Demographic and economic factors [14,17,48,52,53,54,58,67,70,93,95,98,132,133] ○ Nature of the healthcare system [77,91,99,118,119] ○ Technology readiness and innovation mindset [3,30,31,32,57,76,100,120,134,135,136] Policy factors ○ National policy frameworks that promote digitization of healthcare [57,76,77,78,79,82,98,100,102,110,118,119,120,127,128,129,131] ○ Cloud-specific policies [43,44,84,91,92,100,104,120,121] ▪ Data standardization [77,100,106,120] ▪ Data storage [107,108,137,138] ▪ Data privacy [85,91,92,120,134,139] ▪ Cybersecurity [140,141,142,143] ○ Incentives for cloud adoption [80,100,102,103,110,123,127,128,129,136,144,145,146,147] Human factors ○ Public trust and acceptance [101,123,148,149]

**Table 3:** Barriers.

Barriers to Cloud Adoption in Healthcare
Contextual factors ○ Healthcare status [6,81] ○ Technology readiness [125,135,150,151] Organizational factors ○ Cost of adoption [79,83,152] ○ Technical expertise [153,154] Policy factors ○ Lack of data standards [6,74,155,156,157] ○ Strict data storage requirements [107,108,158] ○ Data privacy concerns [135,159] ○ Weak cybersecurity infrastructure [125,160,161]

## Data Availability

Not applicable.

## Abbreviations

AI	Artificial intelligence
CAGR	Compounded annual growth rate
CC	Cloud computing
CD	Compact disk
CDM	Common data module
CHI	Centre for Healthcare Innovation
CHILD	CHI Learning and Development
CiEHR	Center for Interoperable Electronic Health Records
CMIS	Critical Medical Information Store
CSP	Cloud service provider
EHR	Electronic health records
EMR	Electronic medical record
EMRX	Electronic Medical Record Exchange
GDP	Gross domestic product
HAQ	Healthcare Access and Quality
HCNF	Healthcare New Frontier
HIE	Health Information Exchange
ICT	Information and communication technology
IHIS	Integrated Health Information Systems
IoT	Internet of things
KUMC	Korea University Medical Center
MHLW	Ministry of Health, Labor and Welfare
METI	The Ministry of Economy, Trade and Industry
MML	Medical Markup Language
MOH	Ministry of Health
MOHW	Ministry of Health and Welfare
MoKE	Ministry of Knowledge Economy
MSIP	Ministry of Science, Information and Communications Technology and Future Planning
MTCS	Multi-Tier Cloud Security Standard for Singapore
NCB	National Computer Board
NEHR	National Electronic Health Record
NHG	National Health Group
NHIS	National Health Insurance Service
NIST	National Institute of Standards and Technology
NRIC	National Registration Identity Card
NUHS	National University Healthcare System
PDPA	Personal Data Protection Act
PDPC	Personal Data Protection Commission
PHR	Personal health records
PIPA	Personal Information Protection Act
SHVC	Smart Health Video Consultation
SSTN	Social Society and Tax Number
WHO	World Health Organization
